# Supplementary material for: An In Vitro Evaluation of the Capacity of Local Tanzanian Crude Clay and Ash-Based Materials in Binding Aflatoxins in Solution
Source: Toxins (Basel). 2018 Dec 3;10(12):510. doi: 10.3390/toxins10120510 (PMC6316085; doi:10.3390/toxins10120510)
Supplement: Supplementary file 1 [file toxins-10-00510-s001.pdf]

Supplementary Materials: An In Vitro Evaluation of the Capacity of Local Tanzanian Crude Clay and Ash-Based Materials in Binding Aflatoxins in Solution

Emmanuel M. Ayo, Athanasia Matemu, Germana H. Laswai and Martin E. Kimanya

Table S1. Data of the in-vitro test of Tanzanian local binding materials.

1	2	3	4	5	6	7	8	9	10	11	12	13	14	15	16	17	18	19	20
AC	G2	4.140	0.032	4.108	2.773	2.862	2.798	0.024	2.749	2.838	2.774	1.359	1.270	1.335	33.08	30.91	32.49	32.16	1.12
	G1	2.722	0.045	2.677	0.010	0.010	0.010	0.007	0.002	0.002	0.002	2.675	2.675	2.675	99.91	99.91	99.91	99.91	0.00
	B2	4.134	0.073	4.061	1.668	1.678	1.675	0.072	1.596	1.606	1.603	2.465	2.456	2.459	60.69	60.47	60.54	60.57	0.12
	B1	2.975	0.295	2.680	0.077	0.106	0.077	0.029	0.047	0.076	0.047	2.633	2.604	2.633	98.24	97.16	98.24	97.88	0.62
KC	G2	4.140	0.032	4.108	3.543	3.607	3.497	0.035	3.508	3.572	3.462	0.600	0.536	0.646	14.61	13.04	15.72	14.46	1.35
	G1	2.722	0.045	2.677	0.147	0.147	0.147	0.041	0.106	0.106	0.106	2.571	2.571	2.571	96.06	96.06	96.06	96.06	0.00
	B2	4.134	0.073	4.061	2.497	2.508	2.501	0.071	2.426	2.437	2.430	1.635	1.625	1.631	40.25	40.00	40.16	40.14	0.13
	B1	2.975	0.295	2.680	0.153	0.153	0.153	0.030	0.123	0.123	0.123	2.557	2.557	2.557	95.40	95.40	95.40	95.40	0.00
CC	G2	4.140	0.032	4.108	3.478	3.469	3.401	0.052	3.426	3.417	3.349	0.683	0.692	0.759	16.61	16.83	18.47	17.31	1.01
	G1	2.722	0.045	2.677	1.911	1.909	1.851	0.050	1.861	1.859	1.801	0.816	0.818	0.876	30.48	30.55	32.71	31.25	1.27
	B2	4.134	0.073	4.061	3.611	3.494	3.540	0.072	3.539	3.422	3.468	0.523	0.640	0.594	12.87	15.75	14.61	14.41	1.45
	B1	2.975	0.295	2.680	0.456	0.360	0.341	0.293	0.163	0.067	0.048	2.518	2.613	2.632	93.94	97.51	98.22	96.55	2.29
MC	G2	4.140	0.032	4.108	3.134	3.071	3.134	0.043	3.091	3.028	3.091	1.017	1.080	1.017	24.76	26.30	24.76	25.27	0.89
	G1	2.722	0.045	2.677	0.187	0.171	0.200	0.040	0.147	0.131	0.160	2.530	2.546	2.518	94.52	95.10	94.04	94.55	0.53
	B2	4.134	0.073	4.061	2.823	2.819	2.812	0.080	2.743	2.739	2.732	1.318	1.322	1.329	32.46	32.55	32.72	32.58	0.13
	B1	2.975	0.295	2.680	0.148	0.148	0.148	0.029	0.119	0.119	0.119	2.561	2.561	2.561	95.57	95.57	95.57	95.57	0.00
VA	G2	4.140	0.032	4.108	2.920	2.858	2.913	0.052	2.868	2.806	2.861	1.240	1.302	1.247	30.19	31.69	30.35	30.74	0.82
	G1	2.722	0.045	2.677	0.863	0.767	0.793	0.044	0.819	0.723	0.749	1.858	1.954	1.928	69.40	73.00	72.01	71.47	1.86
	B2	4.134	0.073	4.061	2.951	2.998	2.927	0.072	2.879	2.926	2.855	1.182	1.135	1.207	29.10	27.95	29.71	28.92	0.89
	B1	2.975	0.295	2.680	0.353	0.339	0.360	0.295	0.058	0.044	0.065	2.622	2.637	2.615	97.85	98.38	97.57	97.93	0.41
RA	G2	4.140	0.032	4.108	1.146	1.160	1.125	0.022	1.124	1.138	1.103	2.984	2.970	3.005	72.64	72.29	73.16	72.70	0.43
	G1	2.722	0.045	2.677	0.259	0.259	0.276	0.038	0.221	0.221	0.238	2.456	2.456	2.439	91.74	91.74	91.11	91.53	0.36
	B2	4.134	0.073	4.061	0.899	0.892	0.892	0.072	0.827	0.820	0.820	3.234	3.241	3.241	79.64	79.81	79.80	79.75	0.09
	B1	2.975	0.295	2.680	0.186	0.156	0.183	0.030	0.156	0.126	0.153	2.524	2.554	2.527	94.19	95.31	94.29	94.60	0.62
R	G2	4.140	0.032	4.108	0.159	0.199	0.169	0.029	0.130	0.170	0.140	3.978	3.938	3.969	96.83	95.86	96.60	96.43	0.51
	G1	2.722	0.045	2.677	0.047	0.031	0.031	0.004	0.042	0.026	0.026	2.635	2.651	2.651	98.43	99.03	99.03	98.83	0.35
	B2	4.134	0.073	4.061	0.038	0.038	0.038	0.007	0.031	0.031	0.031	4.030	4.030	4.030	99.24	99.24	99.24	99.24	0.00
	B1	2.975	0.295	2.680	0.110	0.081	0.081	0.030	0.080	0.052	0.052	2.600	2.628	2.628	97.02	98.07	98.07	97.72	0.61

Description

1: Types of binding materials.

2: Types of aflatoxin in the test tube.

3: Amount of AF in the test tube with positive control (buffered solution spiked with solution of AF) after incubation.

4: Residual AF (as impurities) in the test tube with negative control (buffers solution without AF spiking); an average of triplicate samples.

5: Actual amount of AF recovered for positive control (3–4).

6, 7, and 8: Amount of AF in the test tube with suspension of binding material in buffer solution spiked with AF solution for triplicate samples 1, 2, and 3, respectively.

9: Residual AF (as impurities) in the test tube with suspension of binding material in buffer solution without AF spiking (blank).

10, 11, and 12: Actual amount of AF in the test tube with suspension of binding material spiked with AF solution; that is, 6–9, 7–9, and 8–9, respectively.

13, 14, and 15: Actual amount of AF bound by binding material after incubation (triplicates); that is, 5–12, 5–13, and 5–14, respectively.

16, 17, and 18: Percent adsorption capacity of the materials in binding AF in buffered solution (triplicates); that is, 13/5*100, 14/5*100, and 15/5*100, respectively.

19: Average percent adsorption capacity of the binding materials in binding AF in buffered solution calculated as mean of the triplicates; that is, (16+17+18)/3.

20: Mean standard deviation of 16, 17, and 18.

AC; Arusha clay, KC; Kilimanjaro clay, CC; Coastal clay, MC; Morogoro clay, VA; Volcanic ash, RA; Rice husk ash, R; Reference binder (Mycobind^{R}).
